# Curriculum of the Irish College of Surgeons, with Strictures

**Published:** 1830-04-01

**Authors:** 


### Curriculum of the Irish College of Surgeons. 1830. With Strictures.

By this Curriculum the system of apprenticeship has received a wound which all the salves and *salvos* of the College will not be able to heal. The system itself had a strong tendency to decay and death; but this Curriculum which, in reality, is designed to prop up a rotten constitution, will only accelerate its dissolution.

The public voice being strong against the apprenticeship system, then, the College has obtained a new charter, in which there are two portals leading to the Temple, one of which is guarded by a dog of the Cerberus species—the other beset with syrens, whose warbling notes are in praise of the old system of apprenticeship and *calculated* to lure all unsuspecting youths to enter through that portal. To approach by the other, the Cerberus is not only to be sopped by very costly dishes, but various gorgons, hydras, and scorpions are planted along the hall, to deter the candidate for collegiate honours from making his way through that entrance. The explanation of this is not very difficult. Candidates for a license may be either registered apprentices or independent students—and that on the following conditions.

"Apprentice.

"Indenture of five years' apprenticeship—Thirty guineas.—Certificates of attendance on hospitals, lectures, dissections, &c. *for a time not defined*!!

"Non-Apprentice.

"Certificates of six years' study.—Sixty guineas.—Certificates of three years, or five winters' hospital attendance, three courses of anatomy and physiology, three of surgery, three of dissections and demonstrations, two of chemistry, one of materia medica, one of practice of medicine, one of midwifery, and one of medical jurisprudence."

The monstrous injustice of leaving undefined the course of study for the apprentice, and which would, doubtless, be construed in his favour by the masters who pocket the profits—coupled with the severe and expensive course of study carefully chalked out for the non-apprentice, and saddled with a double fee to the College, is such, that we would be willing to believe the omission accidental, did we not know that a Bœotion atmosphere surrounds all corporations, and prevents the members from seeing what is clear as noon-day to all other people. Is it not astonishing that men, in the year 1830, could be so stupid as to suppose that the barefaced injustice of the above regulations would not instantly be patent to the meanest capacity, the moment they were read! What is this proceeding altogether but an attempt to prevent the use of a healthy article of intellectual food by a tax designed as a prohibition—while a high premium is offered for the consumption of an article destructive to the mind as poison is to the body? Who can wonder—who indeed can grieve, that radical feelings of insubordination and disaffection should be engendered and flourish, where the medical aristocracy of a country can be guilty of such gross insults on common sense as well as justice and good policy. We shall here introduce the Curriculum and formula of the examinations.

"I. Candidates shall be admitted to an examination for letters testimonial as apprentices, and shall be entitled to the *privileges reserved for apprentices*, if they shall have been duly registered as such on the College books.

"II. Every apprentice so registered shall be admitted to an examination for letters testimonial, if he shall have laid before the Court of Censors the following documents:

"1. A certificate signed by the President, or Vice-President, and two of the Censors, that he has passed an examination as to his acquaintance with the Greek and Latin languages in the following books,—viz. the works of Sallust, the first six books of the Æneid of Virgil, the Satires and Epistles of Horace, the Greek Testament, the Dialogues of Lucian selected by Walker, and the first four books of Homer's Iliad; or a certificate from his tutor that he has entered as a student Trinity College.

"2. His indenture of apprenticeship, with a certificate signed by the member or licentiate to whom he has been indented, that he has fully and perfectly served such apprenticeship for the full term of five years.

"3. A receipt shewing that he has lodged, to the credit of the President, and for the use of the College, in the Bank of Ireland, the sum of thirty guineas.

"4. Certificates of attendance on hospital practice, on lectures on anatomy and physiology, surgery, the practice of medicine, chemistry, materia medica, midwifery, and medical jurisprudence; and of the performance of dissections, and attendance on anatomical demonstrations.

"5. A thesis, essay, or dissertation in Latin or English, fairly engrossed according to a prescribed form, upon any of the following subjects:—Anatomy, physiology, surgery, practice of medicine, chemistry, materia medica, midwifery, or medical jurisprudence; or, in place of such dissertation, a series of cases collected in the hospital in which the candidate has attended, illustrated by comments, or observations.

"III. Every candidate who has not served an apprenticeship shall be admitted to an examination for letters testimonial, if he shall have attended lectures, or hospitals, for three winter seasons at least, in Dublin, London, Glasgow, or Edinburgh; and if he shall have laid before the Court of Censors the following documents:—

"1. A certificate signed by the President, or Vice-President, and two at least of the Censors, that he has passed the examination as to his proficiency in the Greek and Latin languages, as prescribed for the registered apprentices: or a certificate from his tutor that he has entered Trinity College.

"2. A receipt shewing that he has lodged, to the credit of the President, and for the use of the College, in the Bank of Ireland, the sum of sixty guineas.

"3. Certificates shewing that he has been engaged in the study of his profession, in some hospital, or school of surgery, or medicine, for the full term of six years.

"4. Certificates of attendance on a surgical hospital, containing at least 50 patients, during five winter seasons of six months, or three years, if such attendance shall not have been perfected during the winter seasons.

"5. Certificates of attendance on three courses of lectures on anatomy and physiology, three courses of lectures on the theory and practice of surgery, and the performance of three courses of dissections, accompanied by demonstrations; also certificates of attendance on two courses of lectures on chemistry, one course of lectures on materia medica, one course of lectures on the practice of medicine, one course of lectures on midwifery, and one course of lectures on medical jurisprudence.

"6. A thesis, or series of cases, as enjoined for registered pupils.

"IV. No certificate shall be received for attendance on lectures, delivered in Ireland, unless from teachers in schools permitting the visitation of the Court of Censors, and receiving their sanction.

"V. No certificates shall be received from teachers who deliver lectures upon more than one distinct subject, as hitherto allotted to professors in colleges and universities. This regulation, however, shall not exclude the certificates of a teacher who delivers separate, perfect, and distinct courses on anatomy and physiology, and on the theory and practice of surgery.

"VI. No certificate shall be received for attendance on lectures on anatomy and physiology, unless such lectures shall have been delivered upon at least five days in each week of the winter session, between the 1st of October and 1st of May; nor on the theory and practice of surgery, on chemistry, practice of medicine, materia medica, or midwifery, unless delivered within the same period, or at least three days in each week. The two courses delivered in London, and there called autumn and spring courses, shall, however, be considered equivalent to one winter course of six months, as delivered in Dublin and elsewhere.

"VII. Every candidate producing certificates of attendance on lectures, or hospitals, previous to his examination for letters testimonial, shall be liable to be examined respecting their authenticity; and if he shall refuse to answer thereupon, or if it shall appear from his answers, or from any other information obtained by the Court of Censors, that he has not attended such hospital or lectures with regularity, or according to the regulations laid down by the College, he shall not be admitted to an examination for one year; or should it be proved that he has presented a forged certificate, or a certificate obtained by causing some person to personate him and attend for him, he shall never be examined; and if such fraud shall be discovered after the candidate shall have obtained letters testimonial, he shall be expelled, and such letters shall be withdrawn; and shall be given up by him to the College, on demand in writing signed by the Secretary, or, in default thereof, proceedings shall be had against him on his bond.

"VIII. The examination of every candidate for letters testimonial shall be held in the presence of the members and licentiates of the College, or such of them as choose to attend; and the Secretary shall, by regular summonses, give at least four days' notice of such examination.

"IX. Every candidate for letters testimonial shall be solemnly examined on two several days, in anatomy and physiology, in the theory and practice of surgery and medicine, in chemistry, and in materia medica. Candidates shall be expected to perform such surgical operations, or make such dissections on the dead body, as the Court of Censors may require; or they shall be called upon to explain any anatomical preparation which the examiners may lay before them.

"X. In case a candidate shall be rejected, and shall appeal to the Court of Assistants, such appeal, stating that he considers himself aggrieved by the decision of the Court of Censors, shall be lodged within eight days from the date of rejection, and the candidate shall be admitted to examination within fourteen days from the date of such rejection. The examination before the Court of Assistants shall be conducted, in every respect, as the examination before the Court of Censors.

" XI. A candidate for letters testimonial who has been rejected, shall not be admitted to another examination, except upon his appeal, in a less time than one year from such rejection ; and he shall then be required to lay before the Court satisfactory evidence of his attention and opportunities of improvement, subsequent to the period of his rejection.

" XII. The Court of Censors shall be authorized to examine candidates for letters testimonial, who have not been educated in strict conformity with the above by-laws, but who shall produce evidence of having received a professional education equivalent to that required by the College ; provided such candidates apply for an examination, and lodge their certificates and other necessary documents with the Secretary, previous to the 1st of May, 1830. The Court shall also be authorized to receive certificates issued by competent teachers previous to the 1st of May, 1829, notwithstanding any regulation to the contrary in the above by-laws.

" MIDWIFERY DIPLOMA.

" XIII. The mark distinguishing practitioners in midwifery in the printed lists of the College, shall not be affixed to the name of any member or licentiate of the College subsequent to the 1st of November, 1829, unless he shall have obtained the license or diploma of the College, authorizing him to practise that branch of surgery as herein after specified.

" XIV. A Court of Examiners, consisting of a chairman, deputy chairman, and six members, shall be elected by ballot, on the first Monday in January in each year, to examine such members or licentiates as become candidates for the diploma in midwifery ; any four of which court, with the chairman or deputy chairman, shall be competent to hold such examination.

" XV. Every candidate for the diploma in midwifery shall be admitted to an examination, if he shall have laid before the Court the following documents :—

"1. A receipt shewing that he has lodged, to the credit of the President and for the use of the College, in the bank of Ireland, the sum of five guineas.

"2. Certificates of attendance on two courses of lectures on midwifery, of six months' duration each.

"3. A certificate of attendance on an established lying-in hospital for a period of at least six months, or a certificate that he has been a resident pupil for six months in some established dispensary for lying-in women, and diseases of women and children ; such hospital or dispensary to be approved of and sanctioned by the Court.

"4. Satisfactory evidence that he has conducted thirty labour cases at least.

" XVI. Candidates for the midwifery diploma, shall be examined on the anatomy and physiology of the female generative system, the theory and practice of midwifery, and on the diseases of women and children ; and if approved of by the Court, shall receive a license or diploma to that effect, to which the College seal shall be affixed. Should a candidate be rejected, he shall not be admitted again to an examination until a period of three months shall have elapsed, and he shall then be obliged to produce satisfactory evidence of his having been engaged in the study of this branch of surgery subsequent to such rejection.

" WM. AUCHINLECK, President.

" October 23, 1829."

With the detailed regulations affecting the education of the NON-APPRENTICE, we do not find any material fault, provided the same regulations existed respecting the class of *serviles*, for so we must designate those who submit to the degradation of an indenture. But while *their* code of discipline is kept in the dark, and the mysterious words "*privileges reserved for apprentices*," are allowed to blot the page of a public record like this, we must protest against the justice of this College by-law, and consider it a disgrace to the nineteenth century.

---

